# Does the association between physical activity during work and leisure and blood pressure differ across sex? A cross-sectional compositional data analysis in a Danish population-based cohort

**DOI:** 10.1186/s12889-024-20302-5

**Published:** 2024-11-26

**Authors:** Melker S. Johansson, Ole Steen Mortensen, Charlotte Ørsted Hougaard, Michael Hecht Olsen, Mette Korshøj

**Affiliations:** 1 Research Unit of General Practice, Department of Public Health, University of Southern Denmark, JB Winsløws Vej 19, Odense, 5000 Denmark; 2 Department of Sports Science and Clinical Biomechanics, University of Southern Denmark, Campusvej 55, Odense, 5230 Denmark; 3 Department of Occupational and Social Medicine, Part of Copenhagen University Hospital, Holbaek Hospital, Gl. Ringstedvej 4B, Holbaek, 4300 Denmark; 4 Department of Public Health, Section of Social Medicine, University of Copenhagen, Øster Farigmagsgade 5, Copenhagen, 1353 Denmark; 5 Department of Internal medicine, Holbaek Hospital, Smedelundsgade 60, Holbaek, 4300 Denmark; 6 Department of Regional Health Research, University of Southern Denmark, Campusvej 55, Odense, 5230 Denmark

**Keywords:** Occupational physical activity, High blood pressure, Work environment, Prevention of cardiovascular disease, CAMB, Physical activity health paradox

## Abstract

**Background:**

A proposed risk factor for cardiovascular disease is high occupational physical activity (OPA), which seems to increase blood pressure (BP), in contrast to leisure time physical activity. Increased BP may lead to hypertension that increase the risk of cardiovascular disease and premature death. Exposures to OPA differ across sex and also within occupational group. Thus, we aimed to investigate associations between OPA and LTPA and BP among men and women using compositional data analysis.

**Methods:**

This population-based cross-sectional study, used data from the Copenhagen Aging and Midlife Biobank. OPA and LTPA were self-reported time spent in light physical activity (LPA) during work = standing or walking work; moderate-to-vigorous physical activity (MVPA) during work = heavy manual work; LPA during leisure = light physical activity during leisure; MVPA during leisure = biking or walking as commute to work + daily amount of MVPA during leisure, and sleep. Systolic and diastolic BP (SBP, DBP; mmHg) was measured during sitting rest. We used linear regression models to investigate the association between OPA and LTPA, expressed as isometric log-ratios, and BP. The models were used to predict the BP for reallocated physical activity (PA) compositions (i.e., theoretically ‘moving’ time from sitting to PA within each domain). Specifically, we predicted the BP for each reallocated PA compositions and calculated the difference in BP between the reallocated compositions and the mean composition.

**Results:**

In total, 1,334 women and 2,983 men (mean age 55.1 and 52.5 years, respectively) were included in the analyses. About 50% of the women, and 66% of the men, had hypertension. The linear regressions based on the compositional data analysis, showed no association between OPA and LTPA and SBP among women or men. Among men, less time spent sitting and more time spent in LTPA, compared to the mean composition, was associated with a lower DBP (e.g., 60 min less sitting and 60 min more LTPA: -0.25, 95% CI: -0.05, -0.45 mmHg).

**Conclusion:**

No association between OPA and LTPA and BP was observed across sexes, except between LTPA and DBP among men. This could be due to information bias and lack of precision in self-reported time use data of PA.

**Trial registration:**

None.

**Supplementary Information:**

The online version contains supplementary material available at 10.1186/s12889-024-20302-5.

## Background

Globally, hypertension is the leading cause of cardiovascular disease (CVD) and premature death [1, 2]. In 2015, 8.5 million deaths were attributed to a systolic blood pressure (SBP) ≥ 115 mmHg [3]. The majority of individuals with hypertension have essential hypertension [2] (i.e., without a known cause such as another medical condition), yet a variety of factors influence the development and progression of hypertension, as described in the life course perspective [4]. One proposed risk factor is high occupational physical activity (OPA), which has been shown to increase blood pressure (BP) [5–7], which is in contrast to the beneficial effects on BP from leisure time physical activity (LTPA) [5, 8]. These differences between effects on CVD, from OPA and LTPA, may be explained by (1) long periods of increased heart rate due to long bouts of OPA [9, 10] that acutely raise BP [6, 9, 10], (2) insufficient restitution between bouts of high OPA [11], (3) too low intensity of OPA to improve cardiorespiratory capacity [12], and (4) greater amount of static versus dynamic physical activity during OPA [13]. All these mentioned contrasting effects are expected to increase the likelihood of hypertension [6]. Although many strenuous OPA tasks now are performed by use of technical devices, OPA are still present, as indicated by the 42% of workers in Europe stating to almost never sit at work, and 13% stating to spend almost all the worktime in tiring or painful positions [14]. Therefore, studying associations between OPA and BP could reveal a potential for preventing CVD among workers in Europe.

Both the exposure to OPA [15, 16] (across and within occupations [15]) and the onset of CVD differs between men and women [17, 18]. Men seems to be more extensively exposed to OPA, including MVPA and heavy lifting, than women [15, 16, 19]. Also, women seem to hold a biological protection for hypertension until menopause due to estrogen levels [20]. Thus, men may be more susceptible for hypertension due to OPA exposure and biological factors, and it is hence relevant to investigate the associations between OPA and BP among men and women separately.

Time spent in different physical activities are codependent [21]. However, previous studies have estimated the effect of isolated different PA intensities on BP [6, 7], and only one study have accounted for the codependency of time spent in different intensities of PA [22], in relation to BP [5]. The codependency can be accounted for by using compositional data analysis (CoDA) [22], which allows researchers to investigate the effects of physical activities PA on BP from a 24-hour perspective.

This study aimed to investigate associations between PA, during work and leisure, and BP among men and women, separately. We hypothesize that exposure to high levels of OPA will be associated with higher BP, and exposure to high levels of LTPA will be associated with lower BP, especially among men.

## Methods

### Data sources

This population-based cross-sectional study are based on data from Copenhagen Aging and Midlife Biobank (CAMB) collected from 2009 to 2011 [23, 24]. Participants from three previous cohorts (The Metropolit Cohort (10,171 men born in Copenhagen in 1953); The Copenhagen Perinatal Cohort (8,102 men and women born at the National University Hospital in Copenhagen in 1959–1961); and the Danish Longitudinal Study on Work, Unemployment, and Health (11,082 men and women born in 1949 and 1959) [23]), representing a random sample of the Danish population, were invited to answer a questionnaire and participate in a health check. Relevant tests for the current study include resting BP, blood samples, and waist circumference [23, 24]. CAMB was conducted according to the Helsinki declaration and approved by the local ethical committee (H-A-2008-126), and the Danish Data Protection Agency (2008-41-2938).

### Inclusion criteria

CAMB participants were included based on the following criteria: informed consent given, participation in health check including measurement of BP, no diagnosis of ischemic heart disease at baseline, self-reported data on OPA and LTPA, a total sum of self-reported time spent in different physical activities between ≥ 16 - ≤32 h per day and being active at the labor market at baseline. Participants were not encouraged to sum the time spent in the different categories (i.e., to check whether it summed up to 24 h). However, as the exposure of PA are analyzed as compositions (i.e., parts relative to a whole or 100%), the total absolute number of daily hours reported does not influence the analyses as we are investigating the influence of relative time on BP. On the other hand, larger deviations from a reported sum of 24 h may reflect a higher degree of under- or overestimation of one or more physical activities, which result in more measurement error and, thereby, an increased risk of regression dilution bias. Therefore, we investigated whether the inclusion of ≥ 16 - ≤32 h per day affected the results by conducting a sensitivity analysis where we used a stricter inclusion criteria (total sum of 22–26 h).

### Assessment of exposure

Exposure to LTPA and OPA was assessed using a questionnaire. For LTPA, study participants were asked to fill out the daily time (hours and minutes) spent in the following activities: biking or walking to/from work, sedentary behaviour and sleep [23]. Participants were also asked about time spent in light, moderate, and strenuous LTPA. Since these activities were reported as weekly hours and minutes, we calculated daily estimates by dividing the weekly response with 7. For OPA, study participants filled out the daily time (hours and minutes) spent in sitting, standing, walking or heavy manual work [23].

The following categories of PA were used:

- Light physical activity (LPA) during work = standing or walking work;
- Moderate-to-vigorous physical activity (MVPA) during work = heavy manual work;
- LPA during leisure = light PA during leisure;
- MVPA during leisure = biking or walking as commute to work + daily amount of MVPA during leisure.

The majority of the participants spent little time in MVPA during work, which limited relevant time reallocations (part of compositional data analysis, see details below) in the analysis. Therefore, LPA and MVPA, during work and leisure, respectively, were collapsed into PA. The PA composition hence consisted of sitting during leisure and work, respectively, LTPA, OPA, and sleep. In a secondary analysis, participants reporting 0 min in either MVPA or LPA during work and/or leisure were excluded to enable time reallocations between LPA or MVPA and another part of > 1 min. Further, in a secondary analysis, we only included those participants reporting ≥ 1 min of LPA or MVPA during OPA.

### Assessment of outcome

The outcome was resting SBP and diastolic BP (DBP) (mmHg), measured at the health check after at least 10 min sitting rest [23]. The BP was measured 6 times, 3 times at the right arm and 3 times at the left arm while sitting, an average of the 6 measurements was used in the analyses. Only participants with all 6 measurements were included.

*Assessment of potential confounders*.

The following variables were included as potential confounders, as they in previous investigations have been found to significantly affect the associations between PA and BP:

- *age* (calculated from date of birth) [25];
- *occupational factors* (psychosocial work environment, by rating the level of influence at decisions on your own work and the climate among your colleagues [26]; and heavy occupational lifting, reporting exposure to heavy occupational lifting at some point in the entire career) [6],
- *risk factors for CVD* (diabetes, defined as either a self-reported diagnosis of diabetes, register-based use of anti-diabetic medication [dispensed prescription in the Danish National Prescription Registry [27]], or having a measured level of glycated hemoglobin of ≥ 6.5%; smoking, reporting to currently or previously being a smoker [28]; alcohol, current alcohol consumption reported in units of alcohol per week; waist circumference, measurement of the narrowest circumference between the lowest costae and the iliac crest [cm] [29]; menopause, among women only, reporting to presently menstruate [17, 18]; high density lipoprotein (HDL), low density lipoprotein (LDL), triglycerides (TG), and total cholesterol in non-fasting blood samples [23]; hypertension, defined as a self-reported diagnosis with hypertension, register-based use of anti-hypertensive medication, or a measured BP ≥ 140/≥ 90 mmHg);
- *socioeconomic factors* (income, register-based yearly income in Danish kroner [DKK] per person in a family [30]; civil status, reporting to be co-habiting with another adult); and.
- *self-reported capacity* (cardiorespiratory fitness and muscle strength compared to peers, reported on a scale from 1 to 9) [31].

### Statistical analyses

Characteristics of study participants were described using means with standard deviations (SD) for continuous variables and frequencies with percentages (%) for categorical variables, overall and stratified by sex. The PA composition was described using geometric means adjusted to sum up to 24 h. A significance level of 0.05 and corresponding 95% confidence interval (CI) were used in all analyses.

Compositional data exist in a sample space that is not compatible with ‘classical’ statistical methods, such as linear regression. To make this possible, the PA composition was transformed into a set of isometric log-ratio (ilr) coordinates [32, 33]. The response categories for OPA and LTPA did not include reporting durations < 1 min, and participants were not guided towards reporting all minor bouts of PA, such as walking to the coffee machine or climbing a staircase. Thus, not all participants reported MVPA during work. Since compositional parts containing zeros cannot be transformed, we applied a zero-replacement method [34], to be able to include these participants in the analyses. Additionally, a secondary analysis was performed among the participants reporting ≥ 1 min of LPA or MVPA during OPA. Furthermore, were a sensitivity analysis performed only including those participants reporting days of 22–26 h.

In the crude and adjusted linear regression models, the associations between the PA composition and BP were investigated in the following steps: (i) multiple linear regression models were fitted and residuals visually checked, participants with missing covariates were excluded from the analyses (*n* = 1,204); (ii) as the beta-coefficients of the ilr-coordinates are not directly interpretable, we performed pairwise one-to-one time reallocations between sitting and OPA, and LTPA, respectively, to quantify the measure of association. Time reallocations were domain-specific (i.e., no time were reallocated between leisure- and work-activities). Specifically, we predicted the BP for each reallocated PA composition and the mean composition. Subsequently, we calculated the difference in BP (mmHg) between the reallocated compositions and the mean composition. The estimated difference in BP therefore reflects the predicted “effect” of a theoretical time reallocation. Moreover, as both exposure to OPA [15, 16], as well as onset of CVD [17, 18], differs across sex, the analyses were sex-stratified.

### Building of the statistical models

The assumptions of linearity, independence, homoscedasticity, and normal distribution of residuals were checked by visual inspection of residuals vs. continuous variables, residuals vs. fitted values, and quantile-quantile plots of the residuals, respectively. We fitted the following models: Model 1: unadjusted; Model 2: adjusted for age, occupational factors (psychosocial work environment, exposure to occupational lifting), risk factors for CVD (diabetes, smoking, alcohol, waist circumference, lipids, and hypertension), and menopause (women only); Model 3: model 2 + socioeconomic factors (income, civil status) and self-reported capacity (cardiorespiratory fitness, muscle strength). The descriptive statistics were performed in SAS, version 9.4 (SAS Institute, Cary, NC, US). All remaining analyses were performed in RStudio (version 2021.08.10; RStudio Team [2020]. RStudio: Integrated Development for R. RStudio, PBC, Boston, MA. http://www.rstudio.com) running R version 4.1.1 (R Core Team [2022]. R: A language and environment for statistical computing. R Foundation for Statistical Computing, Vienna, Austria. https://www.R-project.org) using the packages “compositions” and “robcompositions”.

## Results

**Fig. 1:**
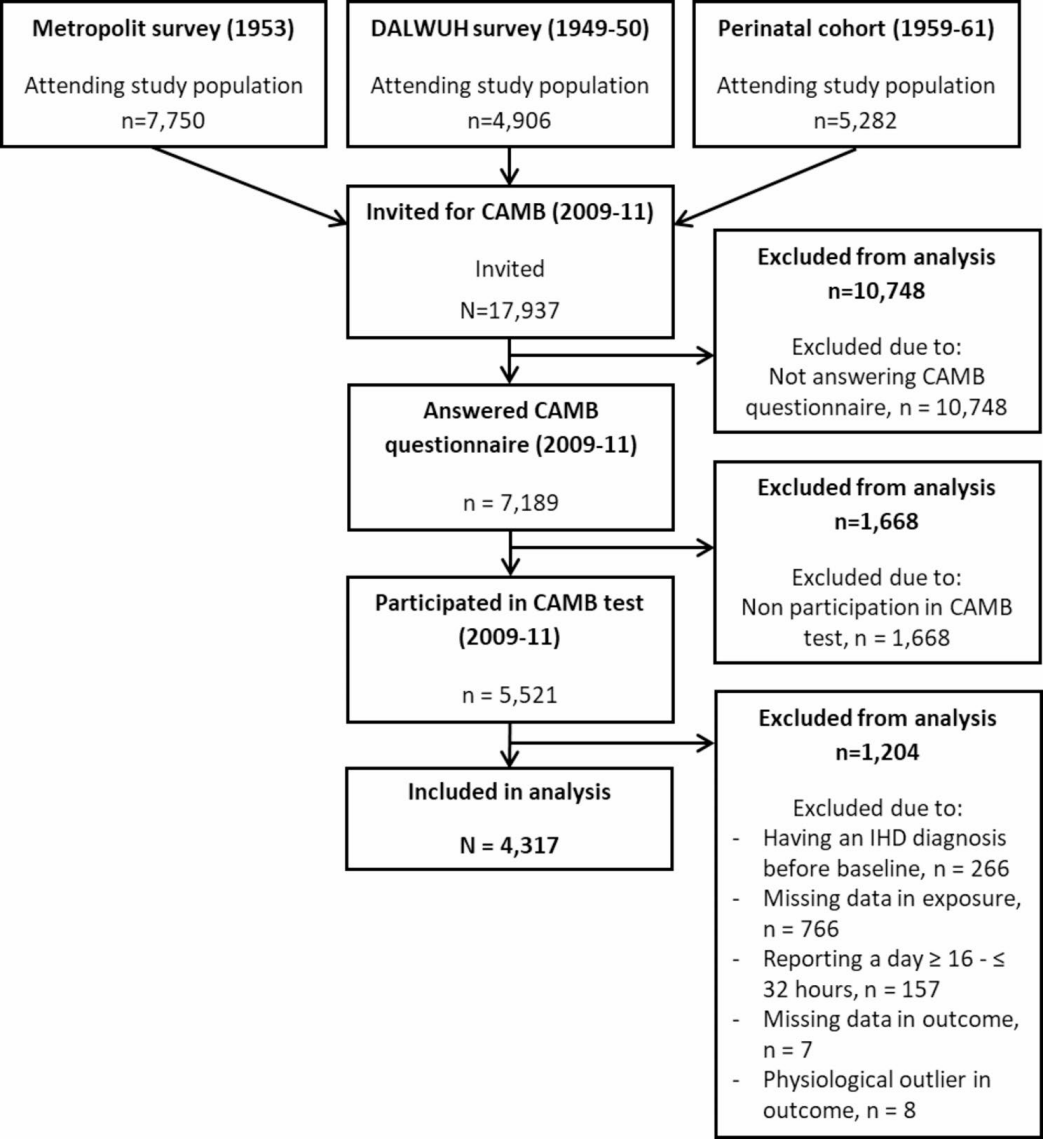
Flow of the participants. DALWUH, the Danish Longitudinal Study on Work, Unemployment, and Health. N/n, number of observations. CAMB, Copenhagen Aging and Midlife Biobank

A total of 4,317 study participants (1,334 women and 2,983 men) were included (Fig. 1). The mean age of the women and men was 55.1 years 52.5 years, respectively. About 50% of women were classified as hypertensives and 66% of the men (Table 1). The population reporting ≥ 1 min of LPA or MVPA during OPA (*n* = 1,303, 434 women and 869 men) differed from the full population by a lower level of income, among both men and women; shorter education among women and longer educated men; a higher level of OPA, including higher levels of exposure to heavy occupational lifting among both men and women; and lower level of LTPA (Supplementary Table 1). These differences are assumed to be attributed by the selection criteria for the population reporting ≥ 1 min of LPA or MVPA during OPA.

**Table 1:** Baseline characteristics of the included participants in the analysis, *N* = 4,317

	ALL, *n* = 4,317		Men, *n* = 2,983		Women, *n* = 1,334	
	Mean (SD)	*n* (%)	Mean (SD)	*n* (%)	Mean (SD)	*n* (%)
Age (years)	54.21 (3.74)		55.14 (3.24)		52.15 (3.96)	
Waist circumference (cm)	95.12 (11.76)		98.23 (10.41)		88.18 (11.65)	
Menopause (%yes among women)						703 (53.10%)
Cardiorespiratory fitness (ml O_2_/min/kg)	33.84 (8.68)		34.87 (9.17)		32.99 (8.16)	
Self-reported cardiorespiratory fitness						
Lower than peers		575 (13.34%)		311 (10.44%)		264 (19.83%)
Same as peers		2046 (47.48%)		1,370 (46.00%)		676 (50.79%)
Higher than peers		1,688 (39.17%)		1,297 (43.55%)		391 (29.38%)
Self-reported muscle strength						
Lower than peers		327 (7.59%)		141 (4.73%)		186 (13.97%)
Same as peers		2,364 (54.86%)		1,598 (53.66%)		766 (57.55%)
Higher than peers		1,618 (37.55%)		1,239 (41.61%)		379 (28.47%)
High density lipoprotein cholesterol (mmol/l)	1.50 (0.40)		1.41 (0.35)		1.71 (0.41)	
Low density lipoprotein cholesterol (mmol/l)	3.03 (0.84)		3.07 (0.83)		2.96 (0.87)	
Triglyceride (mmol/l)	1.75 (1.04)		1.91 (1.12)		1.40 (0.70)	
Total cholesterol (mmol/l)	6.20 (1.11)		6.22 (1.11)		6.16 (1.09)	
Systolic blood pressure (mmHg)	135.20 (17.41)		139.23 (16.17)		126.18 (16.71)	
Diastolic blood pressure (mmHg)	87.97 (10.47)		89.45 (10.15)		84.67 (10.44)	
Blood pressure ≥ 140/≥90 mmHg		2,005 (46.44%)		1,600 (53.64%)		405 (30.36%)
Hypertensive (%yes diagnosted and/or daily use of antihypertensive medicine and/or blood pressure ≥ 140/≥90 mmHg)		2,642 (61.20%)		1,977 (66.28%)		665 (49.85%)
Diabetic (%yes diagnosed and/or daily use of anti-diabetic medicine and/or > 6.5% Hb1Ac		248 (5.74%)		198 (6.64%)		50 (3.75%)
Smoking (% current smokers)		906 (21.00%)		626 (20.99%)		280 (21.01%)
Alcohol consumption (unit/week)	11.77 (11.31)		13.83 (12.26)		7.13 (6.80)	
Education						
No formal education or semi-skilled		417 (9.69%)		303 (10.20%)		114 (8.55%)
Skilled worker		1,473 (34.22%)		1,038 (34.94%)		435 (32.63%)
Short or middle further education		1,590 (36.94%)		988 (33.25%)		602 (45.16%)
Long further education		794 (18.45%)		619 (20.83%)		175 (13.13%)
Other, non-classified		30 (0.70%)		23 (0.77%)		7 (0.53%)
Income (register-based equivalent income DKK/year/person in a family)	342,738.25 (261,716.15)		353,165.30 (298,103.77)		319,445.43 (149,119.09)	
Co-habituating (%yes)		3,514 (82.39%)		2,506 (84.98%)		1,008 (76.60%)

During a 24-hour day, the women and men reported to spend 700 min (49%) and 698 min (48%) in bed (sleep), 114 min (8%) and 104 min (7%) on OPA, 218 min (15%) and 231 min (16%) on occupational sitting, 159 min (11%) and 145 min (10%) on LTPA, and 250 min (17%) and 262 min (18%) on leisure time sitting (Table 2, Supplementary Table 2).

**Table 2:** Baseline characteristics of the physical activity parameters, *N* = 4,317

	ALL, *n* = 4,317		Men, *n* = 2,983		Women, *n* = 1,334	
	Mean (SD)	*n* (%)	Mean (SD)	*n* (%)	Mean (SD)	*n* (%)
Occupational physical activity (number of years)						
Predominantly sedentary	16.67 (13.66)		17.17 (13.59)		15.55 (13.76)	
Sitting or standing, some walking	7.84 (11.28)		7.82 (11.28)		7.86 (11.28)	
Walking, some handling of material	7.66 (11.79)		8.19 (12.34)		6.48 (10.36)	
Heavy manual work	4.42 (9.52)		5.12 (10.25)		2.84 (7.39)	
Occupational heavy lifting (%yes)		1,671 (38.82%)		1,200 (40.32%)		471 (35.44%)
Time spent sedentary (hours/day)	4.63 (2.91)		4.73 (2.96)		4.41 (2.77)	
Time spent standing/walking (LPA) (hours/day)	2.86 (2.53)		2.81 (2.50)		2.96 (2.61)	
Time spent in moderate to vigorous occupational physical activity (MVPA) (hours/day)	0.59 (1.32)		0.65 (1.42)		0.44 (1.04)	
Influence in decisions on own work (%often/always)		3,244 (75.14%)		2,348 (78.71%)		896 (67.17%)
Good collaboration and climate among colleagues (%often/always)		4,122 (95.48%)		2,849 (95.51%)		1,273 (95.43%)
Leisure time physical activity						
Inactive		393 (9.14%)		295 (9.93%)		98 (7.37%)
Light physical active ≥ 4 h/week		2,419 (56.24%)		1,555 (52.32%)		864 (65.01%)
Moderate physical active ≥ 4 h/week		1,386 (32.23%)		1,036 (34.86%)		350 (26.34%)
Vigorous physical activity regularly and several times per week		103 (2.39%)		86 (2.89%)		17 (1.28%)
Time spent sleeping (hours/day)	7.15 (0.81)		7.09 (0.81)		7.27 (0.79)	
Time spent sedentary (hours/day)	2.94 (1.32)		2.97 (1.33)		2.87 (1.29)	
Time spent standing/walking (LPA) (hours/day)	0.79 (0.65)		0.73 (0.62)		0.92 (0.70)	
Time spent in moderate to vigorous leisure time physical activity (MVPA) (hours/day)	0.50 (0.52)		0.53 (0.56)		0.45 (0.41)	

### Associations between blood pressure and physical activity during work and leisure

**Fig. 2:**
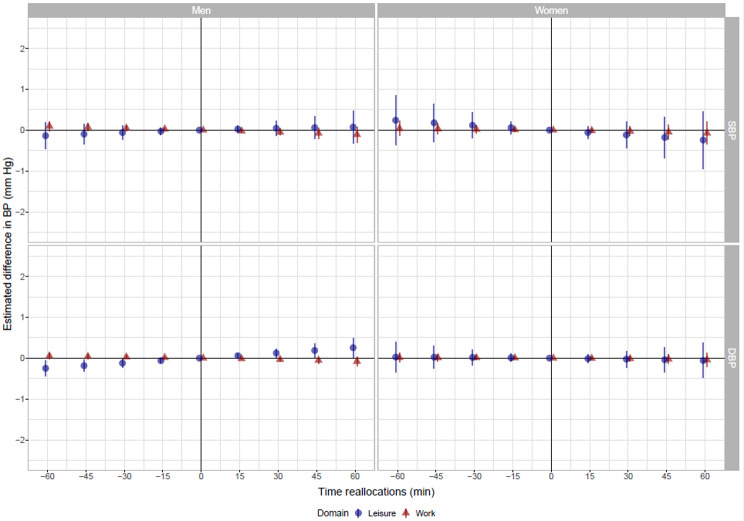
Estimated difference in systolic and diastolic blood pressure (mmHg, y-axis), by the reallocation of time between sitting and physical activity at work and leisure among 1,334 women and 2,983 male participants. The negative reallocations reflect less time spent sitting replaced by more time in physical activity and the positive reallocations reflect more time spent sitting replaced by less time in physical activity. The presented values are adjusted for age, psychosocial work environment, exposure to heavy occupational lifting, diabetes, smoking, alcohol, waist circumference, menopause (among women only), level of high density lipoprotein, low density lipoprotein, triglycerid, total cholesterol, hypertension, income, civil status, self-reported cardiorespiratory fitness and muscle strength

Among women, no associations between OPA and LTPA, and SBP or DBP were found, in either of the models (Fig. 2; Tables 3 and 4). Among men, no associations between OPA and LTPA, and SBP were seen, except in the unadjusted model for the association between LTPA and SBP. The unadjusted model showed lower SBP when reallocating time from sitting to LTPA, and higher SBP when reallocating time from LTPA to sitting. Furthermore, time reallocations during leisure (but not during work) were associated with differences in DBP among men in the fully adjusted model. Specifically, less time spent sitting and more time spent in LTPA was associated with a lower DBP (e.g., reallocating 60 min. from sitting to PA: -0.25 mmHg, 95% CI: -0.05, -0.45; Fig. 2), and more time spent sitting and less time spent in LTPA was associated with a higher DBP (reallocating 60 min. from LTPA to sitting: 0.26 mmHg, 95% CI: 0.02, 0.50; Fig. 2).

None of the estimated differences in SBP or DBP reached a magnitude of clinical relevance. Since the unadjusted estimates did not differ much from those of the fully adjusted models, the unadjusted estimates do not seem to be confounded by traditional CVD risk factors.

**Table 3:** Estimated difference in systolic blood pressure given time reallocations between sitting and physical activity during work and leisure among 1,334 women and 2,983 men. Analyses adjusted for age, psychosocial work environment, exposure to heavy occupational lifting, diabetes, smoking, alcohol, waist circumference, menopause (among women only), level of high density lipoprotein, low density lipoprotein, triglyceride, total cholesterol, hypertension, income, civil status, self-reported cardiorespiratory fitness, and muscle strength

Time reallocations	Domain			
	Occupational Estimated difference in SBP (mmHg) (95% CI) ^#^		Leisure time Estimated difference in SBP (mmHg) (95% CI)	
Sitting and physical activity	Women	Men	Women	Men
-60 min from sitting to physical activity	0.04 (0.22 - -0.14)	0.10 (0.21 - -0.02)	-0.24 (0.85 - -0.37)	-0.14 (0.19 - -0.46)
-45 min from sitting to physical activity	0.03 (0.16 - -0.11)	0.07 (0.16 - -0.02)	0.18 (0.64 - -0.28)	-0.10 (0.15 - -0.34)
-30 min from sitting to physical activity	0.02 (0.11 - -0.07)	0.05 (0.11 - -0.01)	0.12 (0.42 - -0.19)	-0.06 (0.10 - -0.23)
-15 min from sitting to physical activity	0.01 (0.06 - -0.04)	0.02 (0.06 - -0.01)	0.06 (0.21 - -0.10)	-0.03 (0.06 - -0.11)
0 (reference composition)	0.00 (0.00–0.00)	0.00 (0.00–0.00)	0.00 (0.00–0.00)	0.00 (0.00–0.00)
+ 15 min from physical activity to sitting	-0.01 (0.04 - -0.07)	-0.02 (0.01 - -0.06)	-0.06 (0.10 - -0.22)	0.02 (0.11 - -0.06)
+ 30 min from physical activity to sitting	-0.03 (0.08 - -0.14)	-0.05 (0.03 - -0.13)	-0.12 (0.21 - -0.44)	0.05 (0.23 - -0.14)
+ 45 min from physical activity to sitting	-0.05 (0.13 - -0.23)	-0.08 (0.05 - -0.20)	-0.18 (0.32 - -0.68)	0.06 (0.34 - -0.22)
+ 60 min from physical activity to sitting	-0.07 (0.20 - -0.34)	-0.11 (0.08 - -0.30)	-0.24 (0.46 - -0.94)	0.07 (0.47 - -0.32)

^#^Confidence Interval; SBP, systolic blood pressure

**Table 4:** Estimated difference in diastolic blood pressure given time reallocations between sitting and physical activity during work and leisure, adjusted for age, psychosocial work environment, exposure to heavy occupational lifting, diabetes, smoking, alcohol, waist circumference, menopause (among women only), level of high density lipoprotein, low density lipoprotein, triglyceride, total cholesterol, hypertension, income, civil status, self-reported cardiorespiratory fitness and muscle strength. Among the 1,334 women and 2,983 male participants

Time reallocations	Domain			
	Occupational Estimated difference in DBP (mmHg) (95% CI)^#^		Leisure time Estimated difference in DBP (mmHg) (95% CI)	
Sitting and physical activity	Women	Men	Women	Men
-60 min from sitting to physical activity	0.01 (0.12 - -0.10)	0.05 (0.12 - -0.02)	0.03 (0.40 - -0.35)	**-0.25** **(-0.05 - -0.45)**
-45 min from sitting to physical activity	0.01 (0.09 - -0.07)	0.04 (0.09 - -0.01)	0.02 (0.30 - -0.26)	**-0.19** **(-0.04 - -0.33)**
-30 min from sitting to physical activity	0.01 (0.06 - -0.05)	0.03 (0.06 - -0.01)	0.02 (0.20 - -0.17)	**-0.12** **(-0.02 - -0.22)**
-15 min from sitting to physical activity	0.01 (0.03 - -0.02)	0.01 (0.03 - -0.01)	0.01 (0.10 - -0.09)	**-0.06** **(-0.01 - -0.11)**
0 (reference composition)	0.00 (0.00–0.00)	0.00 (0.00–0.00)	0.00 (0.00–0.00)	0.00 (0.00–0.00)
+ 15 min from physical activity to sitting	-0.01 (0.03 - -0.04)	-0.02 (0.01 - -0.04)	-0.01 (0.09 - -0.11)	**0.06** **(0.01–0.11)**
+ 30 min from physical activity to sitting	-0.02 (0.05 - -0.08)	-0.03 (0.01 - -0.08)	-0.02 (0.17 - -0.22)	**0.12** **(0.02–0.23)**
+ 45 min from physical activity to sitting	-0.03 (0.09 - -0.14)	-0.05 (0.02 - -0.13)	-0.04 (0.27 - -0.35)	**0.19** **(0.02–0.36)**
+ 60 min from physical activity to sitting	-0.04 (0.12 - -0.21)	-0.08 (0.04 - -0.20)	-0.06 (0.37 - -0.48)	**0.26** **(0.02–0.50)**

^#^Confidence Interval; DBP diastolic blood pressure

### Secondary analyses

Among the population reporting ≥ 1 min of LPA or MVPA during OPA, during a 24-hour day, the women and men reported to spend 598 min (42%) and 577 min (40%) in bed (sleep), 236 min (16%) and 240 min (17%) on LPA during work, 45 min (3%) and 60 min (4%) on MVPA during work, 201 min (14%) and 205 min (14%) on occupational sitting, 63 min (4%) and 51 min (4%) on LPA during leisure, 74 min (5%) and 75 min (5%) on MVPA during leisure, and 223 min (15%) and 231 min (16%) on leisure time sitting (Supplementary Table 2). Compared to the full population, the population reporting ≥ 1 min of LPA or MVPA during OPA, stated to spend more time during the 24-hour in OPA, less in LTPA, and less time in bed, for both women and men (Supplementary Table 2).

Among both women and men, no associations between OPA and LTPA, and SBP or DBP, were observed, nor did the direction of the associations differ between work and leisure (Supplementary Fig. 1a and b, Supplementary Table 3). One of the estimated differences in SBP from reallocating time between sitting and MVPA, during work, reached a magnitude of clinical relevance among women, although statistically insignificant (Supplementary Table 3), indicating beneficial effects of lowered SBP (reallocating 45 min from MVPA to sitting during work: -2.41 mmHg, 95% CI: 6.05, -10.88; Supplementary Table 3).

### Sensitivity analysis

Among the population reporting a day of 22–26 h (*n* = 564), the women and men reported to spend 646 min (45%) and 626 min (43%) in bed (sleep), 212 min (15%) and 218 min (15%) on OPA, 101 min (7%) and 95 min (7%) on occupational sitting, 197 min (14%) and 195 min (14%) on LTPA, and 284 min (20%) and 306 min (21%) on leisure time sitting (Supplementary Table 2). The sensitivity analysis, only including participants reporting a day of 22–26 h (*n* = 564), showed similar results to the main analyses (Supplementary Tables 5 and 6), both with respect to numerical values, level of significance, and direction of association.

## Discussion

### Summary of findings

No association between either OPA or LTPA and BP was observed among men or women. These results do not support the hypothesis that BP plays an important role in the PA health paradox [5–7, 11], and is not in agreement with previously reported sex-differences in the association between OPA and CVD risk [35]. The results in the current study do not agree with findings from previous studies. For example, studies using accelerometer-based measurements of PA and a CoDA approach have found indications of contrasting associations between OPA and LTPA, and BP; however, these studies did not stratify the analyses by sex [5, 8]. Additionally, studies based on self-reported physical activities [6, 7] and technically measured PA [9, 10], using a ‘traditional’ analytical approach (i.e., non-CoDA), limited to one domain (i.e., either work or leisure), and not stratifying on sex, have also found results in line with the PA health paradox. A possible reason for the disagreement in results could be the use of self-reported PA data in the current study, which are known to be prone to measurement error [36], and thereby increasing the risk of regression dilution bias (i.e., bias the regression coefficient towards the null).

Reducing SBP by 1–5 mmHg is suggested to lower the risk of major cardiovascular events [37, 38]. Likewise, a 1 mmHg increase in resting SBP is shown to be associated with an increased risk for ischemic heart disease mortality by 3.5%, irrespective of the initial level of resting BP [39]. Although statistically significant associations were observed between reallocating time between sitting and LTPA and DBP, the magnitude of these associations were below the clinically relevant level (< 1 mmHg) and should therefore be interpreted as a finding due to chance.

### Strengths and limitations

The participants in the current study are in the end of their working age. Therefore, a presence of healthy worker selection bias is assumed, as those not being able to keep a sufficiently high capacity to keep their physically strenuous job may have either migrated to less physically strenuous jobs or retired. Also, only few of the participants reported high levels of MVPA during work. Thus, the presented results may reflect less hazardous effects from OPA than among a population of more varying age and exposure to MVPA during work. In addition, the BP measurements were conducted in Copenhagen, and the invited participants mostly lived across Zealand. This increase the risk of selection bias, since less healthy participants are less likely to travel for the BP measurement. The included study population would hence be expected to be healthier than the entire invited population and workforce of similar age.

The geometric mean of time in bed was relatively high among both women and men (i.e., 49% and 48%, respectively). This was likely attributed to a lack of reported time spent in all PA and a large variation in the total daily time reported. As aforementioned, the participants were not encouraged to sum the hours of day, which might explain the large variation in the total number of hours of a day. However, as the exposure of PA are analyzed as compositions (i.e., parts relative to a whole or 100%), the total absolute number of daily hours reported does not influence the analyses, as we are investigating the influence of relative time on BP. Importantly, the mean time in bed seems plausible (women 7.27 [SD 0.79] hours per day, men 7.09 [SD 0.81] hours per day, Table 1). However, larger deviations from a reported sum of 24 h may reflect more under- or overestimation (i.e., measurement error) of one or more physical activities by the study participants, which increase the risk of regression dilution bias and null findings. Thus, a sensitivity analysis was conducted, only including participants reporting a day of 22–26 h (*n* = 564). This sensitivity analysis showed results similar, both with respect to numerical values, level of significance, and direction of association, to the main analyses (Supplementary Tables 5 and 6). Thus, the inclusion of participants with ≥ 16 - ≤32 h per day does not seem to have influenced the results.

The data collection did not allow participants to report durations < 1 min and did not explicitly encourage participants to report all minor bouts of PA, such as climbing a staircase. This guidance in the reporting of time use may have introduced information bias, in particular among occupational groups with very short and in-frequent bouts of LPA or MVPA. For example, office workers may not report any LPA or MVPA, despite most likely accumulating time in LPA when walking to the printer or coffee machine, etc., and in MVPA when climbing a staircase. Furthermore, the participants were not encouraged to sum the time spent in the different categories (i.e., to check whether the sum was 24 h). The large variation in the total number of hours of a day (16 to 32) highlights the high degree of measurement error. This has most likely biased the regression estimates towards null (i.e., regression dilution bias), and thus increased the risk for a null finding. On the other hand, the sensitivity analysis among those reporting a day of 22–26 h (i.e., as an attempt to decrease measurement error), as well as the secondary analysis among the population, reporting ≥ 1 min of LPA or MVPA during OPA, showed similar results as the main analysis, which supports a ‘true’ null-finding.

Furthermore, the collection of resting BP during consultation have a lower prognostic value than BP collected during sleep or 24-hours [40, 41], and therefore 24-hour BP measures could also be considered to include in future investigations.

These analyses contribute to the current knowledge by testing whether questionnaire-based data on time spent in PA may be used in CoDA, and which limitations future studies should pay attention to in using such data in CoDA.

## Conclusions

No association of clinical relevance between OPA and LTPA and BP was seen among either women or men. This may be attributed to information bias and measurement error in the self-reported PA measurements leading to regression dilution bias. Future studies investigating associations between OPA and LTPA and BP should use as valid and accurate exposure measurements as possible, such as accelerometer-based or calibrated self-reported PA data.

## Electronic supplementary material

Below is the link to the electronic supplementary material.

Supplementary Material 1

## Data Availability

Data cannot be shared for ethical/privacy reasons, however access to data may be allowed by application to the steering committee for the CAMB cohort, see more info here: https://camb.ku.dk/.

## Abbreviations

BP: Blood pressure
CoDA: Compositional data analysis
CVD: Cardiovascular disease
DBP: Diastolic blood pressure
LTPA: Leisure time physical activity
LPA: Light physical activity
MVPA: Moderate to vigorous physical activity
OPA: Occupational physical activity
SBP: Systolic blood pressure
SD: Standard deviation
