# Supplementary material for: Does the association between physical activity during work and leisure and blood pressure differ across sex? A cross-sectional compositional data analysis in a Danish population-based cohort
Source: BMC Public Health. 2024 Nov 26;24:3290. doi: 10.1186/s12889-024-20302-5 (PMC11600667; doi:10.1186/s12889-024-20302-5)
Supplement: Supplementary file 1 — Supplementary Material 1 [file 12889_2024_20302_MOESM1_ESM.docx]

**supplementary tables**

**Supplementary table 1.** Baseline characteristics of the included participants in the secondary analysis, N=1,303, the high-risk population.

|  | **MEN, n=869** | | **WOMEN, n=434** | |
| --- | --- | --- | --- | --- |
|  | **Mean (SD)** | **n (%)** | **Mean (SD)** | **n (%)** |
| **Age (years)** | 54.89 (3.32) |  | 51.94 (3.80) |  |
| **Waist circumference (cm)** | 98.33 (10.67) |  | 88.51 (11.61) |  |
| **Menopause (%yes among women)** |  |  |  | 235 (55.04%) |
| **Cardiorespiratory fitness (mlO_2_/min/kg)** | 33.75 (8.65) |  | 33.37 (8.04) |  |
| **Self-reported cardiorespiratory fitness** |  |  |  |  |
| **Lower than peers** |  | 85 (9.79%) |  | 86 (19.91%) |
| **Same as peers** |  | 419 (48.27%) |  | 211 (48.84%) |
| **Higher than peers** |  | 364 (41.94%) |  | 135 (31.25%) |
| **Self-reported muscle strenght** |  |  |  |  |
| **Lower than peers** |  | 32 (3.69%) |  | 56 (12.96%) |
| **Same as peers** |  | 426 (49.08%) |  | 235 (54.40%) |
| **Higher than peers** |  | 410 (47.24%) |  | 141 (32.64%) |
| **High density lipoprotein cholesterol (mmol/l)** | 1.42 (0.35) |  | 1.72 (0.41) |  |
| **Low density lipoprotein cholesterol (mmol/l)** | 3.09 (0.83) |  | 2.96 (0.82) |  |
| **Triglyceride (mmol/l)** | 1.95 (1.27) |  | 1.40 (0.70) |  |
| **Total cholesterol (mmol/l)** | 6.22 (1.10) |  | 6.21 (1.04) |  |
| **Systolic blood pressure (mmHg)** | 139.03 (16.36) |  | 127.38 (17.40) |  |
| **Diastolic blood pressure (mmHg)** | 89.26 (10.10) |  | 85.41 (10.34) |  |
| **Blood pressure ≥140/≥90 mmHg** |  | 445 (51.21%) |  | 145 (33.41%) |
| **Hypertensive (%yes diagnosted and/or daily use of antihypertensive medicine and/or blood pressure ≥140/≥90 mmHg)** |  | 559 (64.33%) |  | 224 (51.61%) |
| **Diabetic (%yes diagnosted and/or daily use of anti-diabetic medicine and/or >6.5% Hb1Ac** |  | 61 (7.02%) |  | 18 (4.15%) |
| **Smoking (% current smokers)** |  | 200 (23.01) |  | 104 (23.96%) |
| **Alcohol consumption (unit/week)** | 14.02 (12.87) |  | 7.07 (6.47) |  |
| **Education** |  |  |  |  |
| **No formal education, semi-skilled or other** |  | 125 (14.39%) |  | 44 (10.16%) |
| **Skilled worker** |  | 412 (47.41%) |  | 131 (30.25%) |
| **Short or middle further education** |  | 238 (27.39%) |  | 224 (51.73%) |
| **Long further education** |  | 94 (10.82%) |  | 34 (7.85%) |
| **Socioeconomic status** | 320,290.81 (358,540.24) |  | 317,040.30 (149,930.06) |  |
| **Co-habituating (%yes)** |  | 725 (83.91%) |  | 320 (74.77%) |
| **Occupational physical activity (number of years)** |  |  |  |  |
| **Predominantly sedentary** | 10.70 (11.95) |  | 11.12 (13.39) |  |
| **Sitting or standing, some walking** | 9.35 (11.59) |  | 8.32 (11.01) |  |
| **Walking, some handling of material** | 14.03 (13.80) |  | 10.80 (11.76) |  |
| **Heavy manual work** | 8.22 (11.74) |  | 4.94 (9.27) |  |
| **Occupational heavy lifting (%yes)** |  | 568 (65.66%) |  | 254 (58.66%) |
| **Time spent sedentary (hours/day)** | 3.45 (2.40) |  | 3.39 (2.31) |  |
| **Time spent standing/walking (LPA) (hours/day)** | 3.68 (2.12) |  | 3.71 (2.23) |  |
| **Time spent in moderate to vigorous occupational physical activity (MVPA) (hours/day)** | 1.25 (1.34) |  | 0.96 (1.12) |  |
| **Influence in decisions on own work (%often/always)** |  | 654 (75.26%) |  | 276 (63.59%) |
| **Good collaboration and climate among colleagues (%often/always)** |  | 837 (96.32%) |  | 420 (96.77%) |
| **Leisure time physical activity** |  |  |  |  |
| **Inactive** |  | 65 (7.51%) |  | 26 (6.02%) |
| **Light physical active ≥ 4 hours/week** |  | 460 (53.12%) |  | 301 (69.68%) |
| **Moderate physical active ≥ 4 hours/week** |  | 314 (36.26%) |  | 98 (22.69%) |
| **Vigorous physical activity regularly and several times per week** |  | 27 (3.12%) |  | 7 (1.62%) |
| **Time spent sleeping (hours/day)** | 7.06 (0.85) |  | 7.22 (0.77) |  |
| **Time spent sedentary (hours/day)** | 3.09 (1.28) |  | 2.93 (1.22) |  |
| **Time spent standing/walking (LPA) (hours/day)** | 0.83 (0.64) |  | 0.98 (0.73) |  |
| **Time spent in moderate to vigorous leisure time physical activity (MVPA) (hours/day)** | 0.64 (0.63) |  | 0.48 (0.41) |  |

**Supplementary table 2.** Geometric mean of 24h physical activity compositions among the 2,983 male and 1,334 female participants in the CAMB, and among the 434 female and 869 male participants in the CAMB reporting ≥ 1 min. in either light physical activity (LPA) and/or moderate to vigorous physical activity (MVPA) during occupational physical activity (OPA), stratified by intensity of physical activity and domain, and among the 157 female and 407 male participants reporting days of 22 – 26

|  | **Domain** | | | |
| --- | --- | --- | --- | --- |
| **Physical activity intensity** | **Occupational Minutes (%) of a 24h day** | | **Leisure time Minutes (%) of a 24h day** | |
| **FULL POPULATION**  **N = 4,317** | **Women** | **Men** | **Women** | **Men** |
| **Sitting** | 217.95 (15.14) | 230.96 (16.04) | 249.78 (17.35) | 261.80 (18.18) |
| **Physical activity (PA)** | 113.78 (7.90) | 103.66 (7.20) | 158.64 (11.02) | 145.25 (10.09) |
| **Sleep** |  |  | 699.86 (48.60) | 698.32 (48.49) |
| **POPULATION REPORTING ≥ 1 min IN LPA OR MVPA IN OPA**  **N = 1,303** |  |  |  |  |
| **Sitting** | 201.38 (13.98) | 204.76 (14.22) | 222.58 (15.46) | 231.43 (16.07) |
| **Light physical activity (LPA)** | 236.10 (16.40) | 240.35 (16.69) | 62.86 (4.37) | 51.10 (3.55) |
| **Moderate to vigorous physical activity (MVPA)** | 45.25 (3.14) | 60.19 (4.18) | 74.38 (5.17) | 75.49 (5.24) |
| **Sleep** |  |  | 597.54 (41.50) | 576.69 (40.05) |
| **POPULATION REPORTING DAYS OF 22 – 26 HOURS**  **N = 564** |  |  |  |  |
| **Sitting** | 105.58 (7.33) | 102.42 (7.11) | 296.42 (20.58) | 325.73 (22.62) |
| **Physical activity (PA)** | 212.19 (14.74) | 217.97 (15.14) | 196.98 (13.68) | 194.81 (13.53) |
| **Sleep** |  |  | 674.35 (46.83) | 666.60 (46.29) |

**Supplementary table 3.** Associations between physical activity at work and leisure and systolic blood pressure, adjusted for age, psychosocial work environment, exposure to heavy occupational lifting, diabetes, smoking, alcohol, waist circumference, menopause (among women only), level of high density lipoprotein, low density lipoprotein, triglycerid, total cholesterol, hypertension, income, civil status, self-reported cardiorespiratory fitness and muscle strength. Among the 434 women and 869 male participants, in the population reporting ≥ 1 min. in either light physical activity and/or moderate to vigorous physical activity (MVPA) during occupational physical activity (OPA).

| **Time reallocations** | **Domain** | | | |
| --- | --- | --- | --- | --- |
|  | **Occupational**  **Estimated difference in SBP (mmHg) (95% CI)^#^** | | **Leisure time**  **Estimated difference in SBP (mmHg) (95% CI)** | |
| **Sitting and LPA** | **Women** | **Men** | **Women** | **Men** |
| -45 minutes from sitting to light physical activity | 0.24  (0.25 - -0.72) | 0.14  (0.45 - -0.16) | 0.20  (1.57 - -1.18) | -0.49  (0.42 - -1.39) |
| -30 minutes from sitting to light physical activity | -0.15  (0.16 - -0.47) | 0.10  (0.30 - -0.10) | 0.15  (1.11 - -0.81) | -0.34  (0.30 - -0.98) |
| -15 minutes from sitting to light physical activity | 0.07  (0.08 - -0.23) | 0.05  (0.15 - -0.05) | 0.09  (0.59 - -0.42) | -0.19  (0.16 - -0.53) |
| 0 (reference composition) | 0.00  (0.00 – 0.00) | 0.00  (0.00 – 0.00) | 0.00  (0.00 – 0.00) | 0.00  (0.00 – 0.00) |
| +15 minutes from light physical activity to sitting | 0.07  (0.23 - -0.08) | -0.06  (0.04 - -0.15) | -0.12  (0.49 - -0.73) | 0.23  (0.68 - -0.21) |
| +30 minutes from light physical activity to sitting | 0.14  (0.45 - -0.17) | -0.12  (0.07 - -0.31) | -0.29  (1.13 - -1.70) | 0.57  (1.68 - -0.54) |
| +45 minutes from light physical activity to sitting | 0.21  (0.67 - -0.26) | -0.19  (0.11 - -0.48) | -0.58  (2.10 - -3.25) | 1.31  (3.92 - -1.30) |
| **Sitting and MVPA** |  |  |  |  |
| -45 minutes from sitting to moderate to vigorous physical activity | 0.16  (1.38 - -1.07) | -0.27  (0.32 - -0.86) | 0.02  (1.28 - -1.25) | -0.07  (0.65 - -0.80) |
| -30 minutes from sitting to moderate to vigorous physical activity | 0.13  (1.02 - -0.75) | -0.19  (0.23 - -0.61) | 0.02  (0.89 - -0.86) | -0.04  (0.46 - -0.54) |
| -15 minutes from sitting to moderate to vigorous physical activity | 0.08  (0.57 - -0.41) | -0.10  (0.12 - -0.33) | 0.01  (0.47 - -0.44) | -0.02  (0.24 - -0.28) |
| 0 (reference composition) | 0.00  (0.00 – 0.00) | 0.00  (0.00 – 0.00) | 0.00  (0.00 – 0.00) | 0.00  (0.00 – 0.00) |
| +15 minutes from moderate to vigorous physical activity to sitting | -0.15  (0.52 - -0.82) | 0.13  (0.42 - -0.16) | -0.02  (0.50 - -0.55) | 0.01  (0.31 - -0.29) |
| +30 minutes from moderate to vigorous physical activity to sitting | -0.44  (1.36 - -2.23) | 0.31  (1.00 - -0.38) | -0.06  (1.12 - -1.23) | 0.01  (0.68 - -0.66) |
| +45 minutes from moderate to vigorous physical activity to sitting | -2.41  (6.05 - -10.88) | 0.60  (1.98 - -0.77) | -0.12  (1.94 - -2.18) | -0.004  (1.17 - -1.18) |

^#^Confidence Interval; SBP systolic blood pressure

**Supplementary table 4.** Associations between physical activity at work and leisure and diastolic blood pressure, adjusted for age, psychosocial work environment, exposure to heavy occupational lifting, diabetes, smoking, alcohol, waist circumference, menopause (among women only), level of high density lipoprotein, low density lipoprotein, triglycerid, total cholesterol, hypertension, income, civil status, self-reported cardiorespiratory fitness and muscle strength. Among the 434 women and 869 male participants, in the population reporting ≥ 1 min. in either light physical activity and/or moderate to vigorous physical activity (MVPA) during occupational physical activity (OPA).

| **Time reallocations** | **Domain** | | | |
| --- | --- | --- | --- | --- |
|  | **Occupational**  **Estimated difference in DBP (mmHg) (95% CI)^#^** | | **Leisure time**  **Estimated difference in DBP (mmHg) (95% CI)** | |
| **Sitting and light physical activity** | **Women** | **Men** | **Women** | **Men** |
| -45 minutes from sitting to light physical activity | -0.20  (0.09 - -0.48) | 0.02  (0.20 - -0.16) | -0.13  (0.67 - -0.93) | 0.05  (0.59 – 0.48) |
| -30 minutes from sitting to light physical activity | -0.12  (0.06 - -0.31) | 0.02  (0.14 - -0.10) | -0.08  (0.48 - -0.64) | 0.04  (0.42 - -0.34) |
| -15 minutes from sitting to light physical activity | -0.06  (0.03 - -0.15) | 0.01  (0.07 - -0.05) | -0.04  (0.26 - -0.33) | 0.03  (0.23 - -0.18) |
| 0 (reference composition) | 0.00  (0.00 – 0.00) | 0.00  (0.00 – 0.00) | 0.00  (0.00 – 0.00) | 0.00  (0.00 – 0.00) |
| +15 minutes from light physical activity to sitting | 0.05  (0.14 - -0.04) | -0.01  (0.04 - -0.07) | 0.03  (0.39 - -0.32) | -0.04  (0.22 - -0.31) |
| +30 minutes from light physical activity to sitting | 0.09  (0.27 - -0.09) | -0.03  (0.08 - -0.15) | 0.06  (0.88 - -0.77) | -0.12  (0.54 - -0.78) |
| +45 minutes from light physical activity to sitting | 0.13  (0.41 - -0.14) | -0.05  (0.12 - -0.23) | 0.07  (1.63 - -1.50) | -0.31  (1.24 - -1.86) |
| -45 minutes from sitting to moderate to vigorous physical activity | -0.28  (0.44 - -1.00) | -0.24  (0.11 - -0.59) | 0.10  (0.84 - -0.65) | -0.20  (0.23 - -0.63) |
| -30 minutes from sitting to moderate to vigorous physical activity | -0.18  (0.34 - -0.70) | -0.17  (0.08 - -0.42) | 0.08  (0.59 - -0.43) | -0.14  (0.16 - -0.44) |
| -15 minutes from sitting to moderate to vigorous physical activity | -0.09  (0.20 - -0.37) | -0.09  (0.05 - -0.23) | 0.05  (0.32 - -0.22) | -0.08  (0.08 - -0.23) |
| 0 (reference composition) | 0.00  (0.00 – 0.00) | 0.00  (0.00 – 0.00) | 0.00  (0.00 – 0.00) | 0.00  (0.00 – 0.00) |
| +15 minutes from moderate to vigorous physical activity to sitting | 0.09  (0.48 - -0.30) | 0.11  (0.28 - -0.06) | -0.07  (0.23 - -0.38) | 0.09  (0.27 - -0.09) |
| +30 minutes from moderate to vigorous physical activity to sitting | 0.18  (1.23 - -0.86) | 0.25  (0.66 - -0.16) | -0.19  (0.50 - -0.87) | 0.19  (0.59 - -0.21) |
| +45 minutes from moderate to vigorous physical activity to sitting | 0.40  (5.35 - -4.54) | 0.48  (1.30 - -0.33) | -0.36  (0.84 - -1.57) | 0.34  (1.04 - -0.36) |

^#^Confidence Interval; DBP diastolic blood pressure

**Supplementary table 5.** Associations between physical activity at work and leisure and systolic blood pressure, adjusted for age, psychosocial work environment, exposure to heavy occupational lifting, diabetes, smoking, alcohol, waist circumference, menopause (among women only), level of high density lipoprotein, low density lipoprotein, triglycerid, total cholesterol, hypertension, income, civil status, self-reported cardiorespiratory fitness and muscle strength. Among the 157 women and 407 male participants, reporting a 22 – 26 hour day.

| **Time reallocations** | **Domain** | | | |
| --- | --- | --- | --- | --- |
|  | **Occupational**  **Estimated difference in SBP (mmHg) (95% CI)^#^** | | **Leisure time**  **Estimated difference in SBP (mmHg) (95% CI)** | |
| **Sitting and physical activity** | **Women** | **Men** | **Women** | **Men** |
| -60 minutes from sitting to physical activity | -0.07  (0.86 - -1.00) | -0.15  (0.42 - -0.71) | -0.07  (1.29 - -1.42) | -0.28  (0.42 - -0.97) |
| -45 minutes from sitting to physical activity | -0.06  (0.55 - -0.66) | -0.08  (0.28 - -0.44) | -0.03  (0.98 - -1.05) | -0.20  (0.32 - -0.72) |
| -30 minutes from sitting to physical activity | -0.04  (0.32 - -0.40) | -0.04  (0.17 - -0.26) | 0.01  (0.67 - -0.69) | -0.13  (0.22 - -0.48) |
| -15 minutes from sitting to physical activity | -0.02  (0.14 - -0.19) | -0.02  (0.08 - -0.12) | 0.00  (0.34 - -0.34) | -0.06  (0.11 - -0.24) |
| 0 (reference composition) | 0.00  (0.00 – 0.00) | 0.00  (0.00 – 0.00) | 0.00  (0.00 – 0.00) | 0.00  (0.00 – 0.00) |
| +15 minutes from physical activity to sitting | 0.02  (0.17 - -0.12) | 0.01  (0.10 - -0.08) | -0.01  (0.33 - -0.36) | 0.06  (0.24 - -0.12) |
| +30 minutes from physical activity to sitting | 0.05  (0.32 - -0.22) | 0.01  (0.18 - -0.16) | -0.04  (0.67 - -0.74) | 0.11  (0.48 - -0.25) |
| +45 minutes from physical activity to sitting | 0.08  (0.47 - -0.31) | 0.01  (0.25 - -0.24) | -0.07  (1.01 - -1.15) | 0.17  (0.72 - -0.39) |
| +60 minutes from physical activity to sitting | 0.11  (0.62 - -0.40) | -0.002  (0.32 - -0.32) | -0.12  (1.35 - -1.60) | 0.21  (0.98 - -0.55) |

^#^Confidence interval; SBP systolic blood pressure

**Supplementary table 6.** Associations between physical activity at work and leisure and diastolic blood pressure, adjusted for age, psychosocial work environment, exposure to heavy occupational lifting, diabetes, smoking, alcohol, waist circumference, menopause (among women only), level of high density lipoprotein, low density lipoprotein, triglycerid, total cholesterol, hypertension, income, civil status, self-reported cardiorespiratory fitness and muscle strength. Among the 157 women and 407 male participants, reporting a 22 – 26 hour day.

| **Time reallocations** | **Domain** | | | |
| --- | --- | --- | --- | --- |
|  | **Occupational**  **Estimated difference in DBP (mmHg) (95% CI)^#^** | | **Leisure time**  **Estimated difference in DBP (mmHg) (95% CI)** | |
| **Sitting and physical activity** | **Women** | **Men** | **Women** | **Men** |
| -60 minutes from sitting to physical activity | -0.18  (0.40 - -0.76) | -0.07  (0.28 - -0.43) | -0.01  (0.84 - -0.85) | -0.28  (0.15 - -0.72) |
| -45 minutes from sitting to physical activity | -0.12  (0.25 - -0.50) | -0.04  (0.18 - -0.27) | -0.01  (0.62 - -0.64) | -0.22  (0.11 - -0.54) |
| -30 minutes from sitting to physical activity | -0.08  (0.15 - -0.30) | -0.02  (0.11 - -0.16) | -0.01  (0.41 - -0.43) | -0.15  (0.07 - -0.36) |
| -15 minutes from sitting to physical activity | -0.04  (0.06 - -0.14) | -0.01  (0.05 - -0.07) | -0.004  (0.21 - -0.22) | -0.07  (0.04 - -0.18) |
| 0 (reference composition) | 0.00  (0.00 – 0.00) | 0.00  (0.00 – 0.00) | 0.00  (0.00 – 0.00) | 0.00  (0.00 – 0.00) |
| +15 minutes from physical activity to sitting | 0.04  (0.12 - -0.05) | 0.01  (0.06 - -0.05) | 0.01  (0.22 - -0.21) | 0.08  (0.19 - -0.03) |
| +30 minutes from physical activity to sitting | 0.07  (0.24 - -0.10) | 0.01  (0.12 - -0.09) | 0.01  (0.45 - -0.42) | 0.16  (0.39 - -0.07) |
| +45 minutes from physical activity to sitting | 0.11  (0.35 - -0.14) | 0.01  (0.17 - -0.14) | 0.02  (0.70 - -0.64) | 0.25  (0.60 - -0.10) |
| +60 minutes from physical activity to sitting | 0.14  (0.46 - -0.18) | 0.01  (0.21 - -0.19) | 0.04  (0.95 - -0.88) | 0.35  (0.82 - -0.13) |

^#^Confidence interval; DBP diastolic blood pressure


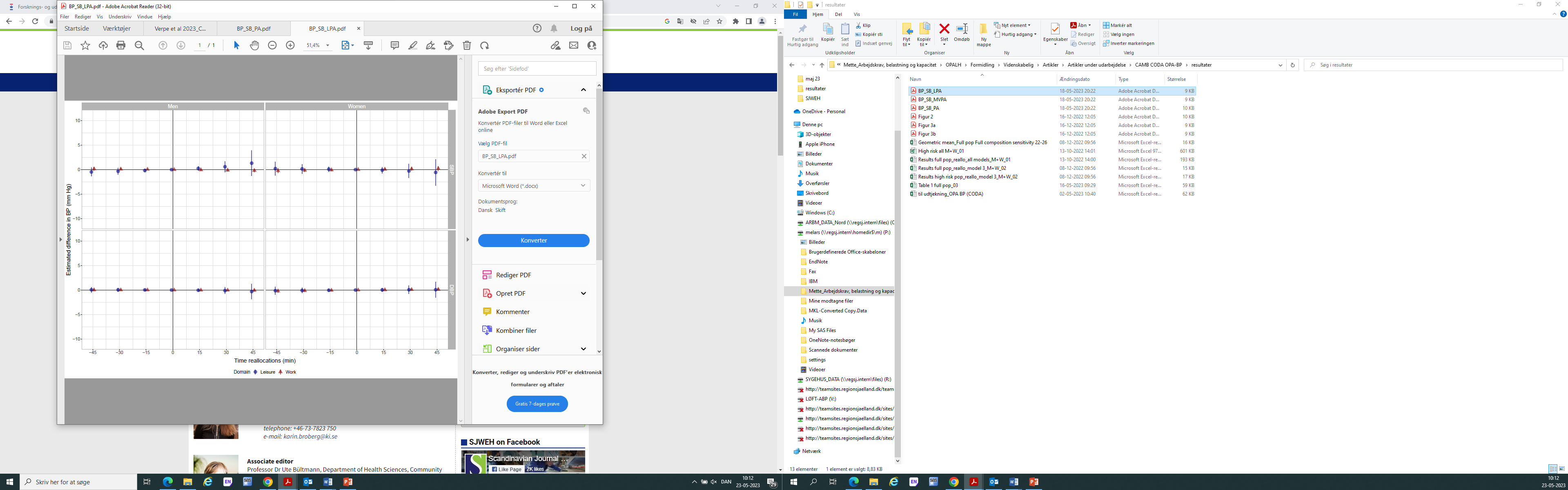


**Supplementary figure 1a.** Estimated level of diastolic and systolic blood pressure (mmHg, y-axis), by the reallocation of time spent sitting and light physical activity at work and leisure, among 434 women and 869 male participants. The negative reallocations reflect less time spent sitting replaced by more time in physical activity and the positive reallocations reflect more time spent sitting replaced by less time in physical activity. The presented values are adjusted for age, psychosocial work environment, exposure to heavy occupational lifting, diabetes, smoking, alcohol, waist circumference, menopause (among women only), level of high density lipoprotein, low density lipoprotein, triglycerid, total cholesterol, hypertension, income, civil status, self-reported cardiorespiratory fitness and muscle strength.


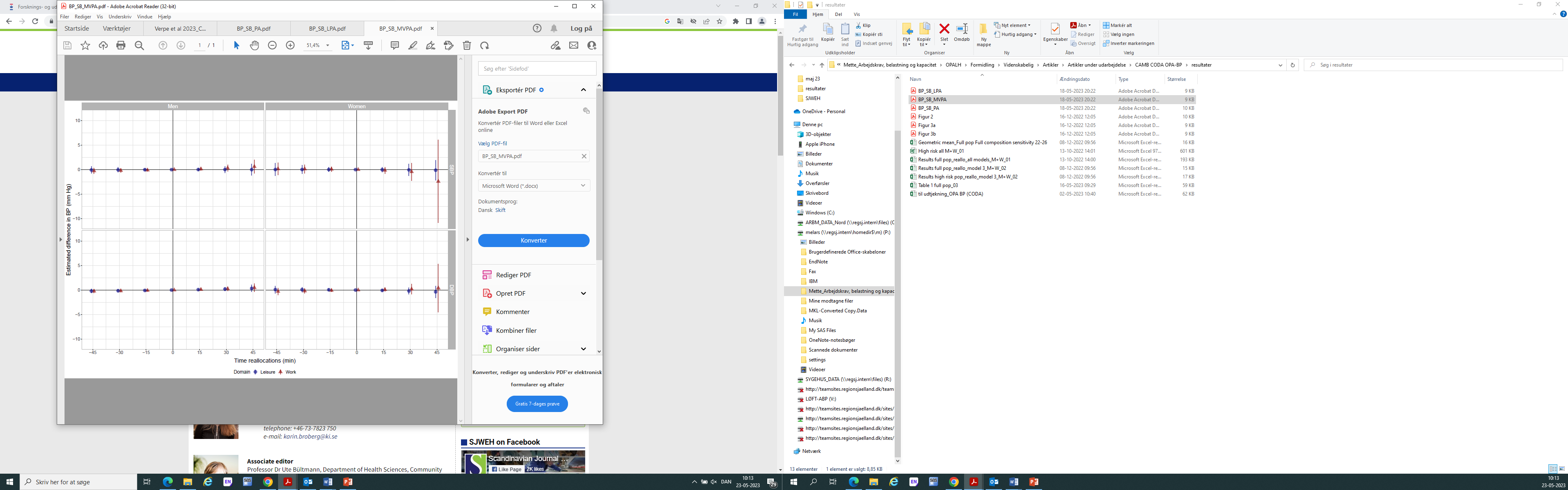


**Supplementary figure 1b.** Estimated level of diastolic and systolic blood pressure (mmHg, y-axis), by the reallocation of time spent sitting and moderate to vigorous physical activity at work and leisure, among 434 women and 869 male participants. The negative reallocations reflect less time spent sitting replaced by more time in physical activity and the positive reallocations reflect more time spent sitting replaced by less time in physical activity. The presented values are adjusted for age, psychosocial work environment, exposure to heavy occupational lifting, diabetes, smoking, alcohol, waist circumference, menopause (among women only), level of high density lipoprotein, low density lipoprotein, triglycerid, total cholesterol, hypertension, income, civil status, self-reported cardiorespiratory fitness and muscle strength.
